# Fucoxantin: A Treasure from the Sea

**DOI:** 10.3390/md10030604

**Published:** 2012-03-07

**Authors:** Nicolantonio D’Orazio, Eugenio Gemello, Maria Alessandra Gammone, Massimo de Girolamo, Cristiana Ficoneri, Graziano Riccioni

**Affiliations:** 1 Human and Clinical Nutrition Unit, Department of Biomedical Science, Via Dei Vestini, University G. D’Annunzio, Chieti, 66013, Italy; Email: egemello@libero.it (E.G.); m.alessandra.gammone@gmail.com (M.A.G.); mdegirolamo@gmail.com (M.G.); bluecris@rocketmail.com (C.F.); griccioni@hotmail.com (G.R.); 2 Cardiology Unit, San Camillo De Lellis Hospital, Manfredonia, FG 71043, Italy

**Keywords:** obesity, fucoxanthin, brown seaweeds, antioxidants, nutrition, fat

## Abstract

The World Health Organization (WHO) estimates that 2.3 billion people will be overweight and 700 million obese in 2015. The reasons for this disastrous trend are attributed to the global tendency toward the reduced magnitude of exercise and physical activity and the increased dietary intake of fats, sugars and calories with reduced amount of vitamins and minerals. To prevent life-style-related diseases, like Metabolic Syndrome (MS), researchers’ attention is increasingly focusing on some of the so called “functional foods” which may be useful for their prevention and treatment. One of these functional ingredients is fucoxanthin (FX), a characteristic carotenoid present in edible brown seaweeds, such as *Undaria pinnatifida *(Wakame), *Hijikia fusiformis* (Hijiki), *Laminaria japonica* (Ma-Kombu) and *Sargassum fulvellum*. The increasing popularity of this molecule is certainly due to its anti-obesity effect, primarily detected by murine studies. These works revealed FX mediated induction of uncoupling protein-1 (UCP-1) in abdominal white adipose tissue (WAT) mitochondria, leading to the oxidation of fatty acids and heat production in WAT. Beyond this important role, in recent studies FX has shown a great antioxidant activity, anti-cancer, anti-diabetic and anti-photoaging properties. The aim of this review is to highlight the main effects of FX on human health.

## 1. Introduction

Cardiovascular diseases (CVD), cancer, and other chronic diseases are nowadays the most frequent causes of death. They all share a multifactorial origin and are caused by a complex interaction between genetic predisposition and personal life style [1]. For this reason an exclusively pharmacological treatment is not always sufficient and, among other factors, nutrition plays a vital contributory or protective role [2]. According to the 2005 survey by the World Health Organization (WHO), globally 1.6 billion people (over 15 years of age) are overweight (body mass index or BMI ≥ 25), and 400 million are obese (BMI ≥ 30). Recently, there has been a rapid increase in the incidence of obesity not only in developed countries such as Europe and USA, but also in developing countries, and it is estimated that in 2015 the number of overweight people will be 2.3 billion and thereof 700 million obese people. WHO reported that the reasons for this disastrous trend are attributed to the global tendency toward the reduced magnitude of exercise and physical activity and the increased dietary intake of fats, sugars and calories with reduced amount of vitamins and minerals. To prevent life-style-related diseases, like the metabolic syndrome (MS), it is important to rectify the poorly balanced nutritional conditions of habitual diet. Researchers are focusing on many functional ingredients in foods which may be useful for the prevention and treatment of life-style-related diseases [3], among them fucoxanthin (FX). 

## 2. Definition and Characterization

FX is a characteristic orange colored carotenoid present in edible brown seaweeds, such as *Undaria pinnatifida *(Wakame), *Hijikia fusiformis* (Hijiki),* Laminaria japonica *(Ma-Kombu) and *Sargassum fulvellum.* It belongs to the class of non-provitamin A carotenoids, a class of 40-carbon organic molecules that consists of two groups: xanthophylls (when their structure contains oxygen) and carotenes (without oxygen in chemical formula). FX is a xanthophyll whose distinct structure includes an unusual allenic bond, epoxide group, and conjugated carbonyl group in polyene chain with antioxidant properties [4,5]. 

Marine algae can be divided into two groups, namely microalgae and macroalgae (seaweeds). There are about 6000 species of seaweed divided into three main classes: green (chlorophytes), red (rhodophytes) and brown (phaeophytes). Brown seaweeds are the major class, contributing approximately to the 59% of the total culture production in 2006, followed by red (40%) and green (*<*1%) seaweeds. Especially brown seaweeds contain many types of bioactive compounds such as omega-3 polyunsaturated fatty acids (PUFAs), polyphenols, polysaccharides, fucosterol, and FX. Algal polyphenols possess many biological activities, including anti-inflammatory, hepatoprotective, anti-tumor, anti-hypertensive and HIV-1 reverse transcriptase activities as well as anti-diabetic activity based on the inhibition of α-glucosidase [6]. Anticarcinogenic effects, apoptosis induction on cancer cells, anti-inflammatory effects, and radical scavenging activity are known biological activities of FX [7,8,9]. The antioxidant properties of carotenoids have been suggested as being the main mechanism by which they afford their beneficial health effects [10]. However, it would be difficult to explain all the physiological effects of carotenoids solely by their antioxidant activity. In the last few years nutrigenomics studies have focused on the exceptional ability of FX in modulating the expression of specific genes involved in cell metabolism [11]. This last feature seems to be the main healthy effect of FX.

## 3. Metabolism

### 3.1. Animal Studies

Recently, absorption and metabolism of FX in mice was investigated. Dietary administered FX was converted to amarouciaxanthin A via fucoxanthinol in mice [12,13]. This metabolic conversion was also observed in human hepatoma cell (HepG2) and required NAD(P)^+^ as cofactor [14]. Dietary FX is hydrolyzed to fucoxanthinol in the gastrointestinal tract by digestive enzymes such as lipase and cholesterol esterase and then converted to amarouciaxanthin A in the liver [15,16]. Thus, these metabolites are considered to be the active forms that exert physiological functions in the body. Amarouciaxanthin A is stored in abdominal white adipose tissue (WAT), fucoxanthinol in other tissues. The bioavailability of these metabolites is required for the proper and safe usage of dietary FX. Recent studies have described the pharmacokinetics of FX and its metabolites, and demonstrated their accumulation after oral administration to mice [17]. Dietary FX undergoes de-acetylation in small intestine becoming fucoxanthinol, which enters the systemic bloodstream through lymphatic duct. Some fucoxanthinol is reduced to amarouciaxanthin A in the liver; the remaining part is accumulated in the tissues [18].

### 3.2. Human Studies

Currently there are few data about pharmacokinetics of FX and its metabolites in human subjects. Recent studies reported that fucoxanthinol was detectable in human plasma after the daily intake of wakame. Data about pharmacokinetics of FX demonstrated that bioavailability and metabolism of fucoxanthinol is higher in humans than in mice [18,19]. Mordenti *et al.* [20] reported that smaller, short-lived animals generally clear drugs from their bodies more rapidly than larger, long-lived animals, and that the pharmacokinetic profile of different species was strikingly different, elimination being most rapid for mice and least rapid for human subjects among the species compared.

FX absorption rate is generally affected by the composition of food matrix. The solubility of FX in soybean oil and in other vegetable oils is very low, while FX can easily dissolve in medium-chain triacylglycerols (MCT) or in fish oil [21]. Antioxidants such as vitamin E are generally used in the products of unstable carotenoids such as astaxanthin and FX to protect against oxidation. However, solubility of FX into vitamin E is very low. It is possible to easily mix FX with vitamin E using MCT as a medium [22]. Thus, the higher anti-obesity effect of FX with MCT in comparison with FX alone, would be due to the increase in FX absorption rate and oxidative stability. When the UCP1 expression in WAT was compared between mice fed with purified FX and seaweed lipids containing FX, the higher UCP1 level was found in the mice fed seaweed lipids, although the FX content was the same in both groups [22]. This suggests that the absorption rate of FX is strongly affected by the presence of other components, especially lipids. 

## 4. Toxicity

Japanese researchers performed single and repeated oral dose toxicity studies of FX in order to evaluate the safety of this carotenoid. In the single dose study, FX was orally administered to male and female mice at doses of 1.000 and 2.000 mg/kg. In the repeated doses study, FX at doses of 500 and 1.000 mg/kg was orally administered for 30 days. In both studies, no mortality and no abnormalities in gross appearance were observed. In the repeated doses study, histological observation revealed no abnormal changes in liver, kidney, spleen and gonadal tissues of any of the FX-treated groups [23].

Another group of researchers tested FX-containing oil extracted from microalga, *Chaetoseros* sp. in rats. Single oral dose and 13-week oral subchronic toxicity studies were conducted. In the single oral dose study, no mortality and no change related to the test material were observed. Thus, the 50% lethal dose of microalgal FX oil is more than 2.000 mg/kg body weight. In the 13-week oral dose study, 0.20 or 200 mg/kg body weight of microalgal FX oil was administered. The FX-administered groups, showed no mortality and no abnormalities. This result suggested that the no observed adverse effect level of FX-containing oil extracted from microalga *Chaetoseros* sp. was 200 mg/kg body weight under the tested subchronic dose condition [24].

## 5. Health Properties

### 5.1. Antioxidant Activity

Oxidation is a normal metabolic process, an inevitable consequence of the energy production in mitochondria. This process removes one of the two electrons from oxygen molecules, forcing them to steal an electron from other molecules, causing oxidative damage [25]. Oxidative stress establishes when there is an imbalance between production of free radicals (FRs) and the body’s ability to neutralize them. Carotenoids have been implicated as important dietary nutrients with antioxidant potential. Firstly, they can quench singlet oxygen. This reaction is attributed mainly to a physical mechanism whereby the excess energy of singlet oxygen is transferred to the long central chain of conjugated double bonds in the carotenoid molecule. The carotenoid with added energy is excited to the triplet state and, upon losing the energy as heat, relaxes to the singlet state without any change in structure [26]. 

Another role of carotenoids as antioxidants is attributed to their scavenging of FRs, which steal an electron from the carotenoid or form an adduct with it. Electron-rich carotenoids are more suitable for reaction with FRs. Consequently, the ability of carotenoids to quench singlet oxygen increases with increasing number of conjugated double bands [27]. FX acts as an antioxidant under anoxic conditions whereas other carotenoids have practically no quenching abilities. Most animal tissues under physiological conditions have low oxygen presence. Furthermore, the typical antioxidants are usually proton donors (ascorbic acid, α-tocopherol, glutathione). FX, on the other hand, donates electron as a part of its free-radical quenching function. A combination of these distinct properties is very rarely found among naturally occurring food-derived compounds [28]. High levels of oxidative stress are common in many diseases including atherosclerosis, Parkinson’s disease, Alzheimer’s disease, acute myocardial infarction, chronic fatigue syndrome and fibromyalgia. These diseases are very different, but share the same biochemical imbalance. Thus, FX could become a new weapon to prevent and treat these diseases.

### 5.2. Anti-Cancer Activity

FRs and oxidative stress are clearly involved in the pathogenesis of cancer disease, so carotenoids, by means of their antioxidant properties, are considered effective anti-cancer molecules. Many studies showed the anti cancer properties of FX especially against prostate cancer, human leukemia, and human colon cancer cell lines [29]. The main mechanism is suggested to be the regulatory effect of FX on biomolecules related to apoptosis and cell cycle. The molecular mechanisms proposed are the FX mediated inhibition of cell cycle by inducing cell cycle arrest and enhancing gap junctional intercellular communication. Recent studies reported the involvement of the caspase pathway and the regulation of *Bcl* expression (*Bcl-2* and *Bcl-xL*) in apoptosis induction by FX [30,31,32].

Recently researchers found that FX induces cell cycle arrest in G2/M phase and apoptosis in human gastric cancer MGC-803 cells with the inhibition of growth in MGC-803 cell by FX resulting from cell cycle arrest and apoptosis. Down-regulation of surviving, CyclinB1 by FX might contribute to the anti-cancer effects of FX, which may reduce *CyclinB1* expression through JAK/STAT signal pathway, sequentially inhibiting proliferation of MGC-803 cells [33].

Moreover FX and fucoxanthinol induced cell growth arrest and apoptotic cell death in primary effusion lymphoma (PEL), a rare type of non-Hodgkin’s lymphoma, at a concentration nontoxic to normal cells and these effects may be associated with functional inhibition of Hsp90 chaperon. These results suggest that dietary FX or fucoxanthinol could be potentially helpful for the treatment of PEL [34].

### 5.3. Anti-Obesity Activity

The increasing incidence of obesity is becoming one of most important medical problems. The identification of substances that can decrease or prevent obesity remains a main goal of medical research. Adaptive thermogenesis by uncoupling protein-1 (UCP1) could be a physiological defense against obesity [35]. UCP1 expression is known to be a significant component of whole body energy expenditure, and its dysfunction contributes to the development of obesity [36]. 

During normal metabolism the body produces heat. FX increases the amount of energy released as heat in fat tissue, a process also called thermogenesis. UCP1 induction by FX metabolites accumulated in WAT is of great interest, because, UCP1 is normally expressed only in brown adipose tissue (BAT) and not in WAT. This protein, situated in the mitochondrial inner membrane, dissipates the pH-gradient generated by oxidative phosphorylation, releasing chemical energy as heat. UCP1 expression in WAT by FX intake leads to oxidation of fatty acids and heat production in WAT [37]. 

UCP families are found in many types of tissue such as BAT (UCP1, UCP2 and UCP3), WAT (UCP2), skeletal muscle (UCP2 and UCP3), and brain tissue (UCP4 and UCP5) [38]. *UCP1* gene expression is increased by cold, adrenergic stimulation, β3-agonists, retinoids and thyroid hormone. This adaptive thermogenesis by UCP1 induction in BAT plays an important role in energy balance by dissipating excess energy intake as heat to resist body weight gain. UCP1 expression in BAT is known as a significant component of whole body energy expenditure, at least in small rodents, and its dysfunction contributes to the development of obesity. In contrast to rodents, humans have not great amounts of BAT. Considered as a breakthrough discovery for an ideal therapy for obesity, the regulation of UCP1 expression in tissues other than BAT by food constituents would also be important. FX was found to induce both protein and mRNA expression of UCP1 in WAT [39]. This finding will give a clue for new dietary anti-obesity therapies. FX intake promotes mRNA expression of β3-adrenergic receptor (Adrb3) in WAT of obese model mice [40]. Adrb3 is mainly expressed in BAT and WAT. The up-regulation of Adrb3 expression is considered to be responsible for lipolysis and thermogenesis. Thus FX metabolites may promote sensitivity to sympathetic nerve stimulation and the up-regulation of fat oxidation in WAT. Another study showed the regulatory effect of FX metabolites on peroxisome proliferator-activated receptor γ (PPARγ) expression in adipose tissue, which is an important modulator for UCP1 expression [41].

All these promising scientific findings have been obtained through animal studies, and therefore the FX, to keep its promises as an anti-obesity nutraceutical, needs to be extensively tested on humans. Only one study has been conducted in humans which has evaluated the effectiveness of FX supplementation for weight loss. In this work Abidov *et al.* [42] tested the FX in 151 non-diabetic, obese premenopausal women. Three quarters of participants were affected by nonalcoholic fatty liver disease (NAFLD), while the remaining had a normal liver function. The women were divided into two groups and invited to take respectively 600 mg of Xanthigen, which contains 300 mg pomegranate seed oil (PSO) and 300 mg brown seaweed extract containing 2.4 mg FX or a placebo for 16 weeks. The diet was reduced to 1800 kcal per day and was composed of 50% carbohydrates, 30% protein and 20% fat. The results provided a significant reduction of body weight, fat and systolic/diastolic blood pressure; decreased levels of TG and of some enzymes (C-reactive protein (CRP), glutamic pyruvic transaminase (GPT), glutamic oxaloacetic transaminase (GOT), γ-glutamyl transpeptidase (γGT), and significant increase in resting energy expenditure (REE) measured by indirect calorimetry. The 16-week supplementation with 4.0 mg/day FX showed an important increase in REE and an even greater increase in the group taking FX at a dose of 8 mg [34]. Obese patients with NAFLD commonly present elevated markers of liver inflammation and injury, including CRP, GOT, GPT and γGT [43]. A significant reduction in body weight and fat in obese individuals results in the down-regulation of inflammatory markers and prevent MS. It has been demonstrated that increased GPT and CRP plasma levels are associated with decreased hepatic insulin sensitivity, insulin resistance and an increased risk for the onset of MS and type 2 diabetes. Judging from this human trial, the FX levels of several brown seaweed lipids will be high enough to show their physiological activity when these materials are applied to general foods.

### 5.4. Antidiabetic Activity

The potential antidiabetic effects of FX are attributable to the ability of this molecule to induce weight loss and WAT reduction. The adipocyte has recently been recognized as an endocrine cell for its role in the secretion of biologically active mediators, termed adipokines/chemokines, including leptin, adiponectin, resistin, tumor necrosis factor-α (TNF-α) and monocyte chemoattractant protein-1 (MCP-1). Some adipokines are reported to alter insulin sensitivity and glucose and lipid metabolism in muscle, liver and adipose tissues [44]. The participation of macrophages in inflammatory responses by the release of pro-inflammatory mediators (TNF-α and MCP-1) under obesity conditions has also been reported. The chronic low-grade inflammation elicited by pro-inflammatory mediators in the WAT leads to insulin resistance [45]. FX and its metabolites prevent the development of diabetes in a model of obese/diabetic mice (KK-Ay), but not in lean C57BL/6J mice through down-regulation of MCP-1, TNF-α, IL-6 and PAI-1 mRNA expression in the WAT of KK-Ay mice fed FX. The regulatory effect of FX on glucose transporter 4 (GLUT4) expression, found in muscle of normal mice fed a high-fat diet, is also related to the antidiabetic effect of FX. Skeletal muscle accounts for nearly 40% of body mass, and its role in the insulin-induced stimulation of glucose uptake is well documented [46]. The administration of a FX-containing diet promoted the recovery of blood glucose uptake to muscle by the up-regulation of GLUT4 mRNA expression. Another marked effect of FX on adipocytokine secretion was a significant decrease in plasminogen activator inhibitor-1 (PAI-1) mRNA expression in WAT of KK-Ay mice [47]. The PAI-1 mRNA level is over-expressed in obese adipose tissue. Increase in PAI-1 has been reported to be linked not only to thrombosis and fibrosis but also to obesity and insulin resistance. In addition, FX and fucoxanthinol affect peroxisome proliferator-activated receptor γ (PPARγ) and promote gene expression related to lipid metabolism in adipocytes [48]. The results of a recent work, using cultivated cells, showed that fucoxanthinol prevents inflammation and insulin resistance also by inhibiting nitric oxide (NO) and PGE2 production through the down-regulation of iNOS and COX-2 mRNA expression as well as adipocytokine production in WAT. iNOS is an enzyme that produces NO, which is a free radical molecule related to the pathogenesis of inflammation. The over-expression of iNOS mRNA has been observed in WAT of obese mice and adipocytes [49]. Treatment of diabetic *db/db* mice with an iNOS inhibitor reversed hyperglycaemia and improved insulin sensitivity, showing that the down-regulation of iNOS mRNA in macrophages is the molecular target for the prevention of not only inflammation but also type-2 diabetes. COX-2 is an inducible enzyme that produces prostaglandin E2 (PGE2), another inflammatory mediator. Pharmacological inhibition of COX-2 reduces IL-6 in WAT, which induces insulin resistance. 

### 5.5. Activity on Cardiovascular System

Because MS is a collection of risk factors that substantially increase the chances of damage in the cardiovascular system (CVS), which can lead to a heart attack or stroke, FX would be very important to prevent CV damage. FX acts on the reduction of major cardiovascular risk factors (obesity, diabetes, high blood pressure, chronic inflammation, plasma and hepatic triglyceride, and cholesterol concentrations) [50,51]. An interesting metabolic benefit of FX administration in rodents is the promotion of the synthesis of docosahexaenoic acid (DHA) in the liver, resulting in improvements in lipid profile [52]. Experiments on stroke-prone spontaneously hypertensive rats (SHRSP) show the possible protective role of FX in CVD. Thirty-three male SHRSP rats, 5 weeks of age, were divided into three groups: (1) kaolin group, which was given a normal diet (kaolin is a non-nutrient material); (2) Wakame (Undaria Pinnatifida) group (normal diet containing Wakame powder); and (3) cellulose group (normal diet containing cellulose). In this study, Wakame delayed the incidence of stroke signs and increased the life span of SHRSP [53]. Clinical research also indicated that the metabolic boost from taking FX did not stimulate the central nervous system, meaning it did not cause jitters or lost sleep like caffeine, nicotine, or thyroid hormones. So the FX may have a potential role in the modulation and prevention of human diseases, particularly in reducing the incidence of CVD [54].

### 5.6. Anti-Photoaging Activity

A recent work showed for the first time the protective effect of FX against UVB-induced skin photoaging. In this study, Japanese researchers examined the effect of FX on UVB-induced photoaging in the skin of hairless mice by evaluating various parameters of photoaging. The results showed that the topical application of FX suppresses UV-induced expression in the skin. The antioxidant activity of FX might be involved in this anti-angiogenic effect, because ROS stimulate angiogenesis [55]. The sunscreen action of FX does not play a major role in its protective effect against UV-induced photoaging, because the UVB absorption (290–320 nm) of FX is relatively weak. This finding might be useful in the exploitation of FX in cosmetics. However the details of the molecular mechanism of the antiangiogenic effect of FX in UV-exposed skin should be addressed in order to determine the mechanism of anti-photoaging [56].

## 6. Conclusions

The versatile effects of FX make this carotenoid of great potential value in the prevention or management of MS, obesity and other chronic diseases. The promising results of animal studies should encourage researchers to undertake human clinical trials, since we currently have little information about the correct dosage of FX and its use in preventing and treating specific diseases. 

As a carotenoid, FX is a powerful antioxidant that protects cells from FRs damage. A diet rich in FX could help to reduce body fat accumulation and to modulate blood glucose and insulin levels, through the regulation of cytokine secretions from WAT. FX proved safe with no side effects, and even provided other health benefits, including improved cardiovascular health, reduction of inflammation (a major cause of heart disease), healthy cholesterol and triglycerides levels, improvements in blood pressure levels, and healthy liver function [57]. Moreover it has proved to be an effective chelator of heavy metals and toxics [58,59]. These findings indicate the functionality of FX rich brown algae as an anti-obesity and anti-diabetic functional food [60].

Since chemical synthesis of FX is possible but very expensive, the possibility of obtaining this precious carotenoid directly from brown seaweeds should not be underestimated. Many brown seaweeds contain a sufficient amount of FX, and are rich in vitamins, minerals, dietary fibers, proteins, PUFAs, polysaccharides, other carotenoids, phlorotannins and various functional polyphenols. Thanks to their content of iodine, they can be used as activator of thyroid function. Moreover, thanks to their content in alginates and mucilage, these algae have the ability to reduce appetite and even the intestinal absorption of carbohydrates and fats thereby having a synergistic action with FX [61,62,63].

## References

[1] Hu F.B., Liu Y., Willett W.C. (2011). Preventing chronic diseases by promoting healthy diet and lifestyle: Public policy implications for China. Obes. Rev..

[2] Kuipers R.S., de Graaf D.J., Luxwolda M.F., Muskiet M.H., Dijck-Brouwer D.A., Muskiet F.A. (2011). Saturated fat, carbohydrates and cardiovascular disease. Neth. J. Med..

[3] Beppu F., Niwano Y., Tsukui T., Hosokawa M., Miyashita K. (2009). Single and repeated oral dose toxicity study of fucoxanthin (FX), a marine carotenoid, in mice. J Toxicol Sci..

[4] Mercadante A.Z., Egeland E.S., Britton G., Liaaen-Jensen S., Pfander H. (2004). Carotenoids with a C40 Skeleton. Carotenoids—Handbook.

[5] Hu T., Liu D., Chen Y., Wu J., Wang S. (2010). Antioxidant activity of sulfated polysaccharide fractions extracted from *Undaria pinnitafida in vitro*. Int. J. Biol. Macromol..

[6] Pallela R., Na-Young Y., Kim S.K. (2010). Anti-photoaging and photoprotective compounds derived from marine organisms. Mar. Drugs.

[7] Yu R.-X., Hu X.-M.., Xu S.-Q., Jiang Z.-J., Yang W. (2011). Effects of fucoxanthin on proliferation and apoptosis in human gastric adenocarcinoma MGC-803 cells via JAK/STAT signal pathway. Eur. J. Pharmacol..

[8] Ayyad S.E., Ezmirly S.T., Basaif S.A., Alarif W.M., Badria A.F., Badria F.A. (2011). Antioxidant, cytotoxic, antitumor, and protective DNA damage metabolites from the red sea brown alga *Sargassum* sp. Pharmacogn. Res..

[9] Rocha F.D., Soares A.R., Houghton P.J., Pereira R.C., Teixeira V.L. (2007). Potential cytotoxic activity of some Brazilian seaweeds on human melanoma cells. Phytother. Res..

[10] Seifried H.E., Anderson D.E., Fisher E.I., Milner J.A. (2007). A review of the interaction among dietary antioxidants and reactive oxygen species. J. Nutr. Biochem..

[11] Miyashita K. (2009). Function of marine carotenoids. Forum Nutr..

[12] Sangeetha R.K., Bhaskar N., Divakar S., Baskaran V. (2010). Bioavailability and metabolism of fucoxanthin in rats: Structural characterization of metabolites by LC-MS (APCI).. Mol. Cell. Biochem..

[13] Sugawara T., Baskaran V., Tsuzuki W., Nagao A. (2002). Brown algae fucoxanthin is hydrolyzed to fucoxanthinol during absorption by Caco-2 human intestinal cells and mice. J. Nutr..

[14] Das S.K., Hashimoto T., Kanazawa K. (2008). Growth inhibition of human hepatic carcinoma HepG2 cells by fucoxanthin is associated with down-regulation of cyclin D. Biochim. Biophys. Acta.

[15] Asai A., Sugawara T., Ono H., Nagao A. (2004). Biotransformation of fucoxanthinol into amarouciaxanthin A in mice and HepG2 cells: Formation and cytotoxicity of fucoxanthinmetabolites. Drug Metab. Dispos..

[16] Asai A., Yonekura L., Nagao A. (2008). Low bioavailability of dietary epoxyxanthophylls in humans. Br. J. Nutr..

[17] Hashimoto T., Ozaki Y., Taminato M., Das S.K., Mizuno M., Yoshimura K., Maoka T., Kanazawa K. (2009). The distribution and accumulation of fucoxan mthin and its metabolites after oral administration in mice. Br. J. Nutr..

[18] Matsumoto M., Hosokawa M., Matsukawa N., Hagio M., Shinoki A., Nishimukai M., Miyashita K., Yajima T., Hara H. (2010). Suppressive effects of the marine carotenoids, fucoxanthin and fucoxanthinol on triglyceride absorption in lymph duct-cannulated rats. Eur. J. Nutr..

[19] Hashimoto T., Ozaki Y., Mizuno M., Yoshida M., Nishitani Y., Azuma T., Komoto A., Maoka T., Tanino Y., Kanazawa K. (2011). Pharmacokinetics of fucoxanthinol in human plasma after the oral administration of kombu extract. Br. J. Nutr..

[20] Mordenti J. (1986). Man *versus* beast: Pharmacokinetic scaling in mammals. J. Pharm. Sci..

[21] Maeda H., Hosokawa M., Sashima T., Miyashita K. (2007). Dietary combination of fucoxanthin and fish oil attenuates the weight gain of white adipose tissue and decreases blood glucose in obese/diabetic KK-Ay mice. J. Agric. Food Chem..

[22] Maeda H., Hosokawa M., Sashima T., Funayama K., Miyashita K. (2007). Effect of medium-chain triacylglycerols on anti-obesity effect of fucoxanthin. J. Oleo Sci..

[23] Beppu F., Niwano Y., Tsukui T., Hosokawa M., Miyashita K. (2009). Single and repeated oral dose toxicity study of fucoxanthin (FX), a marine carotenoid, in mice. J. Toxicol. Sci..

[24] Iio K., Okada Y., Ishikura M. (2011). Single and 13-week oral toxicity study of fucoxanthin oil from microalgae in rats. Shokuhin Eiseigaku Zasshi.

[25] Glaeser J., Nuss A.M., Berghoff B.A., Klug G. (2011). Singlet oxygen stress in microorganisms. Adv. Microb. Physiol..

[26] Roehrs M., Valentini J., Paniz C., Moro A., Charão M., Bulcão R., Freitas F., Brucker N., Duarte M., Leal M. (2011). The relationships between exogenous and endogenous antioxidants with the lipid profile and oxidative damage in hemodialysis patients. BMC Nephrol..

[27] Sachindra N.M., Sato E., Maeda H., Hosokawa M., Niwano Y., Kohno M., Miyashita K. (2007). Radical scavenging and singlet oxygen quenching activity of marine carotenoid fucoxanthin and its metabolites. J. Agric. Food Chem..

[28] Yan X., Chuda Y., Suzuki M., Nagata T. (1999). Fucoxanthin as the major antioxidant in *Hijikia fusiformis*, a common edible seaweed. Biosci. Biotechnol. Biochem..

[29] Kotake-Nara E., Kushiro M., Zhang H., Sugawara T., Miyashita K., Nagao A. (2001). Carotenoids affect proliferation of human prostate cancer cells. J. Nutr..

[30] Hosokawa M., Kudo M., Maeda H., Kohno H., Tanaka T., Miyashita K. (2004). Fucoxanthin induces apoptosis and enhances the antiproliferative effect of the PPARγ ligand, troglitazone, on colon cancer cells. Biochim. Biophys. Acta.

[31] Kotake-Nara E., Asai A., Nagao A. (2005). Neoxanthin and fucoxanthin induce apoptosis in PC-3 human prostate cancer cells. Cancer Lett..

[32] Kim K.N., Heo S.J., Kang S.M., Ahn G., Jeon Y.J. (2010). Fucoxanthin induces apoptosis in human leukemia HL-60 cells through a ROS-mediated Bcl-xL pathway. Toxicol. in Vitro.

[33] Yu R.X., Hu X.M., Xu S.Q., Jiang Z.J., Yang W. (2011). Effects of fucoxanthin on proliferation and apoptosis in human gastric adenocarcinoma MGC 803 cells via JAK/STAT signal pathway. Eur. J. Pharmacol..

[34] Yamamoto K., Ishikawa C., Katano H., Yasumoto T., Mori N. (2011). Fucoxanthin and its deacetylated product, fucoxanthinol, induce apoptosis of primary effusion lymphomas. Cancer Lett..

[35] Jezek P. (2002). Possible physiological roles of mitochondrial uncoupling proteins. Int. J. Biochem. Cell Biol..

[36] Labruna G., Pasanisi F., Fortunato G., Nardelli C., Finelli C., Farinaro E., Contaldo F., Sacchetti L. (2011). Sequence analysis of the UCP1 gene in a severe obese population from southern Italy. J. Obes..

[37] Okada T., Mizuno Y., Sibayama S., Hosokawa M., Miyashita K. (2011). Antiobesity effects of Undaria lipid capsules prepared with scallop phospholipids. J. Food Sci..

[38] Dalgaard L.T., Pedersen O. (2001). Uncoupling proteins: Functional characteristics and role in the pathogenesis of obesity and type II diabetes. Diabetologia.

[39] Maeda H., Hosokawa M., Sashima T., Miyashita K. (2007). Dietary combination of fucoxanthin and fish oil attenuates the weight gain of white adipose tissue and decreases blood glucose in obese/diabetic KK-Ay mice. J. Agric. Food Chem..

[40] Maeda H., Hosokawa M., Sashima T., Murakami-Funayama K., Miyashita K. (2009). Anti-obesity and anti-diabetic effects of fucoxanthin on diet-induced obesity conditions in a murine model. Mol. Med. Rep..

[41] Maeda H., Hosokawa M., Sashima T., Takahashi N., Kawada T., Miyashita K. (2006). Fucoxanthin and its metabolite, fucoxanthinol, suppress adipocyte differentiation in 3T3-L1 cells. Int. J. Mol. Med..

[42] Abidov M., Ramazanov Z., Seifulla R., Grachev S. (2010). The effects of Xanthigen in the weight management of obese premenopausal women with non-alcoholic fatty liver disease and normal liver fat. Diabetes Obes. Metab..

[43] Heilbronn L.K., Noakes M., Clifton M.P. (2001). Energy restriction and weight loss on very-low-fat diets reduce C-reactive protein concentrations in obese, healthy women. Atheroscler. Thromb. Vasc. Biol..

[44] Shoelson S.E., Lee J., Goldfine A.B. (2006). Inflammation and insulin resistance. J. Clin. Invest..

[45] Matsuzawa Y., Shimomura I., Kihara S., Funahashi T. (2003). Importance of adipocytokines in obesity-related diseases. Horm. Res..

[46] Wellen K.E., Hotamisligil G.S. (2005). Inflammation, stress, and diabetes. J. Clin. Invest..

[47] Hosokawa M., Miyashita T., Nishikawa S., Emi S., Tsukui T., Beppu F., Okada T., Miyashita K. (2010). Fucoxanthin regulates adipocytokine mRNA expression in white adipose tissue of diabetic/obese KK-Ay mice. Arch. Biochem. Biophys..

[48] Maeda H., Hosokawa M., Sashima T., Takahashi N., Kawada T., Miyashita K. (2006). Fucoxanthin and its metabolite, fucoxanthinol, suppress adipocyte differentiation in 3T3-L1 cells. Int. J. Mol. Med..

[49] Nozaki M., Fukuhara A., Segawa K., Okuno Y., Abe M., Hosogai N., Matsuda M., Komuro R., Shimomura I. (2007). Nitric oxide dysregulates adipocytokine expression in 3T3-L1 adipocytes. Biochem. Biophys. Res. Commun..

[50] Jeon S.M., Kim H.J., Woo M.N., Lee M.K., Shin Y.C., Park Y.B., Choi M.S. (2010). Fucoxanthin-rich seaweed extract suppresses body weight gain and improves lipid metabolism in high-fat-fed C57BL/6J mice. Biotechnol. J..

[51] Shiratori K., Okgami K., Ilieva I., Jin X.H., Koyama Y., Miyashita K., Yoshida K., Kase S., Ohno S. (2005). Effects of fucoxanthin on lipopolysaccharide-induced inflammation *in vitro* and *in vivo*. Exp. Eye Res..

[52] Park H.J., Lee M.K., Park Y.B., Shin Y.C., Choi M.S. (2010). Beneficial effects of *Undaria pinnatifida* ethanol extract on diet-induced-insulin resistance in C57BL/6J mice. Food Chem. Toxicol..

[53] Ikeda K., Kitamura A., Machida H., Watanabe M., Negishi H., Hiraoka J., Nakano T. (2003). Effect of *Undaria pinnatifida* (Wakame) on the development of cerebrovascular diseases in stroke-prone spontaneously hypertensive rats. Clin. Exp. Pharmacol. Physiol..

[54] Riccioni G., D’Orazio N., Franceschelli S., Speranza L. (2011). Marine carotenoids and cardiovascular risk markers. Mar. Drugs.

[55] Yasuda M., Ohzeki Y., Shimizu S., Naito S., Ohtsuru A., Yamamoto T., Kuroiwa Y. (1999). Stimulation of *in vitro* angiogenesis by hydrogen peroxide and the relation with ETS-1 in endothelial cells. Life Sci..

[56] Urikura I., Sugawara T., Hirata T. (2011). Protective effect of fucoxanthin against UVB-induced skin photoaging in hairless mice. Biosci. Biotechnol. Biochem..

[57] Tsukui T., Konno K., Hosokawa M., Maeda H., Sashima T., Miyashita K. (2007). Fucoxanthin and fucoxanthinol enhance the amount of docosahexaenoic acid in the liver of KKAy obese/diabetic mice. J. Agric. Food Chem..

[58] Perugini M., D’orazio N., Manera M., Giannella B., Zaccaroni A., Zucchini M., Giammarino A., Riccioni G., Ficoneri C., Amorena M. (2006). Total mercury in fish from the Central Adriatic Sea in relation to levels found in the hair of fishermen. J. Vet. Pharmacol. Ther..

[59] Zaccaroni A., Perugini M., D’orazio N., Manera M., Giannella B., Zucchini M., Giammarino A., Riccioni G., Ficoneri C., Naccari C. (2006). Investigation of total arsenic in fish from the Central Adriatic Sea (Italy) in relation to levels found in fishermen’s Hair. J. Vet. Pharmacol. Ther..

[60] Woo M.N., Jeon S.M., Shin Y.C., Lee M.K., Kang M.A., Choi M.S. (2009). Anti obese property of fucoxanthin is partly mediated by altering lipid-regulating enzymes and uncoupling proteins of visceral adipose tissue in mice. Mol. Nutr. Food Res..

[61] Tvrzicka E., Kremmyda L.S., Stankova B., Zak A. (2011). Fatty acids as biocompounds: Their role in human metabolism, health and disease—A review. Part 1: Classification, dietary sources and biological functions. Biomed. Pap. Med. Fac. Univ. Palacky Olomouc Czech. Repub..

[62] Wijesinghe W.A., Jeon Y.J. (2011). Exploiting biological activities of brown seaweed Ecklonia cava for potential industrial applications: A review. Int. J. Food Sci. Nutr..

[63] Maeda H., Tsukui T., Sashima T., Hosokawa M., Miyashita K. (2008). Seaweed carotenoid, fucoxanthin, as a multi-functional nutrient. Asia Pac. J. Clin. Nutr..

